# Low Serum IL-17A in Pregnancy During Second Trimester Is Associated With an Increased Risk of Subclinical Hypothyroidism

**DOI:** 10.3389/fendo.2020.00298

**Published:** 2020-05-14

**Authors:** Leqi He, Xiuju Zhu, Qian Yang, Xiaoying Li, Xinmei Huang, Chunmei Shen, Jun Liu, Bingbing Zha

**Affiliations:** ^1^Department of Clinical Laboratory Medicine, Shanghai Fifth People's Hospital, Fudan University, Shanghai, China; ^2^Department of Endocrinology, Shanghai Fifth People's Hospital, Fudan University, Shanghai, China

**Keywords:** IL-17A, Th17 cells, thyroid stimulating hormone, thyroid function, subclinical hypothyroidism, the second trimester, pregnancy

## Abstract

**Problem:** Interleukin-17A (IL-17A) has a role in sustaining normal pregnancy. IL-17A is also associated with thyroid autoimmunity during pregnancy. This study sought to investigate whether IL-17A is a risk factor for thyroid dysfunction during pregnancy in women negative for thyroid autoantibodies.

**Methods of Study:** The study comprised 216 pregnant women with negative thyroid peroxidase antibody (TPOAb) and thyroglobulin antibody (TGAb) during the second trimester who provided blood samples for serum IL-17A, thyroid autoantibodies and thyroid function tests. To further evaluate the ratio of CD4+IL-17A+ Th17 cells, we collected peripheral blood from 26 women with thyroid-stimulating hormone (TSH) levels ≤ 2.5 mIU/L and 26 pregnancy-week matched women with TSH levels >2.5 mIU/L, along with samples from 20 women with TSH levels ≤ 4 mIU/L and 20 pregnancy-week matched women with TSH levels >4 mIU/L.

**Results:** The serum IL-17A levels and ratios of CD4+IL-17A+ cells were significantly lower in women with TSH > 2.5 mIU/L than in those with TSH ≤ 2.5 mIU/L (both *P* < 0.01). Similar lower differences were noted in women with TSH > 4 mIU/L than in those with TSH ≤ 4 mIU/L (both *P* < 0.01). Moreover, serum TSH correlated negatively with IL-17A levels (β = −0.195, *P* = 0.004), but positively with the week of gestation (β = 0.284, *P* < 0.001). Logistic regression indicated that a lower serum IL-17A level was a risk factor for TSH > 2.5 mIU/L [OR = 0.453 (0.298–0.689), *P* = 0.000] and TSH > 4.0 mIU/L [OR = 0.588 (0.385–0.899), *P* = 0.013].

**Conclusion:** A low serum IL-17A level during the second trimester is associated with an increased risk of TSH > 2.5 mIU/L and subclinical hypothyroidism.

## Introduction

During a successful pregnancy, the maternal immune system must be immunologically tolerant to the semi-allograft fetus by suppressing the maternal alloreactive response against paternal antigens. It is well-known that regulatory T cell (Treg) is the CD4^+^ T cell that maintains maternal-fetal immune tolerance by preventing immune and autoimmune responses against self-antigens. T helper (Th) cells are effector cells that also play an important role in modulating the activity of immune cells through the release of cytokines. The cytokine interleukin-4 (IL-4) secreted by TH2 appear to be favorable for a successful pregnancy (1) compared with the deleterious cytokine tumor necrosis factor-α (TNF-α) secreted by TH1 cells, which is associated with recurrent spontaneous abortion (2, 3). Recent studies have demonstrated that the cytokine milieu at the maternal-fetal interface is vital for the establishment and maintenance of pregnancy.

IL-17-producing T cells, named T helper 17 (Th17) cells, have been characterized as a distinct lineage of CD4^+^ T cells that differentiate from naive T-cell precursors. Transforming growth factor-β (TGFβ), IL-6, and IL-1β are essential elements that initiate Th17 cell development, whereas IL-23 serves as a pivotal factor that drives both the differentiation and inflammatory functions of pathogenic Th17 cells (4). There are six members of the Th17 cytokine family, including IL-17A (commonly referred to as IL-17), IL-17B, IL-17C, IL-17D, IL-17E, and IL-17F. Among all of the members, the biological function and regulation of IL-17A are best understood. IL-17A protects against inflammation during pathogen infection. However, the role that IL-17A plays in tolerating the allogeneic fetus is more controversial. IL-17A has been found in the placentas of healthy pregnancies (5). Th17 cells inhibit the apoptosis of human trophoblast cells by secreting IL-17A during the first trimester of pregnancy (6). IL-17A expression in the human placenta may play a key role in the establishment of pregnancy. However, an excess of IL-17A has been reported in the blood of pregnant women with pre-eclampsia (7) and in the blood and decidua of pre-term birth (8) and recurrent abortion cases (9, 10). The passive transfer of purified Th17 cells into pregnant mice dramatically increases the proportion of fetal loss (11).

Thyroid disease is a common clinical problem in pregnant women (12). The prevalence of subclinical hypothyroidism during the second trimester of pregnancy is 5.96% in China (13). An elevated maternal TSH concentration has been reportedly associated with pregnancy loss, premature delivery, and adverse neurocognitive outcomes in offspring (14–16). Turhan et al. found that serum IL-17A levels in untreated thyroid autoimmunity (TAI) pregnant patients (with high levels of TSH, TPOAb, and TGAb) were significantly higher than those of the control group in the first trimester, but IL-17A levels were similar between euthyroid TAI patients treated with L-T4 and normal pregnant women in the second trimester (17). In fact, IL-17A expression plays a key role in the establishment of pregnancy in addition to the thyroid autoimmune reaction. However, few studies have focused on the relationship between the IL-17A cytokine and thyroid status in pregnant women, excluding the effects of thyroid autoimmunity. Our objective was to investigate whether IL-17A is a risk factor for thyroid dysfunction in the absence of thyroid autoimmunity and levothyroxine interventions in China pregnant women.

## Materials and Methods

### Study Population

The study was performed in Minhang District, Shanghai, an iodine-sufficient area in China (18). We extracted data from pregnant women who were screened for thyroid disease as part of their routine evaluation in the obstetric outpatient department of the Fifth People's Hospital of Fudan University from April 2017 to September 2017. Recruitment criteria included urban residence, age between 18 and 40 years, singleton pregnancy, and residing in an iodine-sufficient area. The following exclusion criteria were applied: (1) chronic infectious disease; (2) positivity of TPOAb and TGAb in the second trimester; (3) multiple pregnancy; (4) a history of thyroid disease; (5) chronic autoimmune disease; (6) gestational diabetes; (7) taking drugs that could affect thyroid function or receiving thyroid hormone replacement therapy; and (8) subclinical and/or overt hyperthyroidism diagnosed during second trimester. A total of 325 pregnant women during the second trimester (gestational age ranging between 13 and 24 weeks, calculated on the basis of the last menstrual period) were initially enrolled in the study, and the following patients were excluded: 17 participants with chronic viral hepatitis; 22 participants with positive TPOAb or TGAb; 8 participants with multiple pregnancy; 10 patients with history of thyroid disease or receiving thyroid hormone replacement therapy; 6 patients with chronic autoimmune disease; and 46 patients with gestational diabetes at gestational week 24. Ultimately, 216 pregnant women were enrolled in our study.

**Figure 1:**
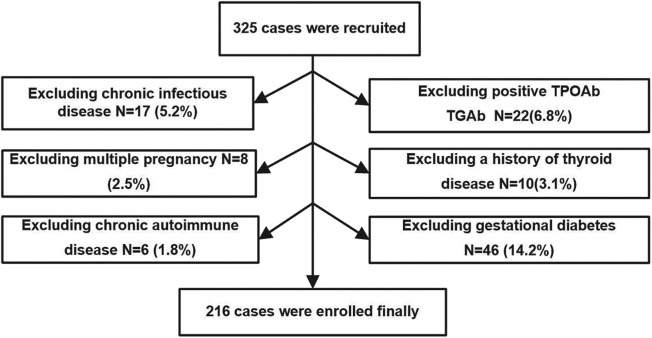
Flow diagram of the procedure used to select subjects.

The procedure used to select the subjects is shown in Figure 1. All studies involving human participants were performed in accordance with the ethical standards of the institutional and/or national research committee and with the Helsinki Declaration. The study protocols were approved by the Medical Ethics Committee of the Fifth People's Hospital of Shanghai, Fudan University (No. 2016-081). Informed consent was obtained from all individual participants included in the study.

### Blood Sampling and Biochemical Parameters

All venous blood samples were collected in the morning after an overnight fast of >8 h from September 2017 to April 2017. If patients was diagnosed with subclinical hypothyroidism (SCH), the blood samples were taken before L-T4 administration. All samples were centrifuged to obtain serum and sent to the clinical laboratory for testing or storage in a serum bank at −80°C until analysis. At the same time, a complete blood count (CBC) was determined using an automated hematology analyzer XE 2100 D (Sysmex Diagnostics, Japan); CBC analyses included the white blood cells, neutrophils, lymphocytes, eosinophils, basophils, monocytes, red blood cells, hemoglobin, and platelets. Creatinine (Cr), alanine aminotransferase (ALT), and fasting blood glucose (FBG) were measured using a MODULAR P 800 automated chemical analyzer (Roche Diagnostics, Germany). The intra-assay coefficients of variation (CVs) of Cr, ALT, and FBG were 3.11–4.07%, 3.31–4.95%, and 2.11–3.25%, respectively. The inter-assay CVs were 3.07–3.49%, 3.16–4.74%, and 2.04–2.39%, respectively.

### Thyroid Function Test and Anti-thyroid Antibodies

SCH is defined as TSH levels >4 mIU/L and a normal free T4 levels (14). The levels of free thyroxine (FT4) (reference range, 12–22 pmol/L), TSH (reference range, 0.27–4.2 mIU/L), TPOAb (cutoff level, 34 IU/mL), and TGAb (cutoff level, 115 IU/mL) were determined in all subjects on the same day of sampling using a commercial euglobulin clot lysis assay (ECLA) kit (ROCHE Diagnostics, cobas Elecsys 601, Germany). The intra-assay CVs for the above measurements were 1.73–2.51%, 1.73–2.44%, 4.07–7.13%, and 4.20–6.60%, respectively. We do not have gestational specific thyroid function reference ranges in our clinical laboratory.

### Cytokine Quantification

TNF-α, IL-4, IL-17A, IFNγ, IL10, IL22, TGFβ, and IL23 were accurately quantified in duplicate using sandwich enzyme-linked immunosorbent assay (ELISA) kits purchased from BioLegend Inc., USA (LEGEND MAXTM kits). The normal concentrations of TNF-α, IL-4, IL-17A, IFNγ, IL10, IL22, TGFβ, and IL23 were 3.5–200 pg/ml, 0.6–250 pg/ml, 0.8–1,000 pg/ml, 5.6–1,000 pg/mL, 3.15–200 pg/mL, 31.3–2,000 pg/mL, 0.23–100 pg/mL, 15.6–2,000 pg/mL, respectively. The variability of the duplicates was <10% of the mean value. According to the manufacturer's instructions and each assay with a standard curve, the values were recorded at wavelengths of 450 and 570 nm and were then calculated from the standard curve. The TNF-α, IL-4, and IL-17A ELISA results had intra-assay CVs of 2.3–10.8%, 4.5–5.3%, 5.4–5.6% and inter-assay CVs of 5.0–6.7%, 6.9–7.1%, 7.8–9.1%, respectively.

### Flow Cytometry

We categorized participants based on different TSH cutoff values (2.5 and 4 mIU/L). To further evaluate the ratio of CD4^+^IL-17A^+^ cells, the flow cytometry analysis was performed in subgroups. We collected peripheral blood from 26 women with TSH levels ≤ 2.5 mIU/L and 26 pregnancy-week matched women with TSH levels >2.5 mIU/L, along with samples from 20 women with TSH levels ≤ 4 mIU/L and 20 pregnancy-week matched women with TSH levels >4 mIU/L.

We collected and diluted 2 ml of peripheral blood by adding an equal volume of 0.9% NaCl. We carefully layered the diluted blood over 3 ml of Lymphoprep™ in a 15-mL centrifuge tube. Mononuclear cells were removed from the interface using a Pasteur pipette to a 24-well cell culture plate. We added 2.5 μl of phorbol myristate acetate (PMA, 10 ng/mL; Sigma Chemical Co., Deisenhofen, Germany) in the presence of brefeldin/monensin for 4–6 h to stimulate the mononuclear leukocytes, which were cultured in RPMI containing 10% FCS for 4 h at 37°C in a humidified atmosphere containing 5% CO_2_. The cells were washed and then stained for 20 min at room temperature with CD3-FITC and CD4-PerCP monoclonal antibodies (eBioscience, San Diego, CA, USA) in the dark. The cells were then washed with PBS (500 μl) and centrifuged at 400 × g for 5 min at room temperature before being treated with permeabilizing solution buffer (BD Bioscience, San Jose, CA, USA) for 20 min at room temperature. They were then stained with PE-conjugated anti-IL-17A (e-Bioscience, San Diego, CA, USA) for 30 min at room temperature. After being washed once, the cells were analyzed using a FACS Calibur flow cytometer and Cell Quest software (BD Bioscience). An isotype-matched PE-conjugated mouse IgG1 antibody (eBioscience) was used as a control.

### Statistical Analysis

According to a significantly higher pregnancy loss rate in TPOAb-negative women with TSH > 2.5 mIU/L group than in those with TSH ≤ 2.5 mIU/L group (19), and SCH (14), we categorized participants into two groups based on different TSH level cutoff values: TSH ≤ 2.5 mIU/L and TSH > 2.5 mIU/L groups, TSH ≤ 4 mIU/L and TSH > 4 mIU/L groups.

The mean ± SD was calculated for normally distributed variables, whereas the median values were determined for data with skewed distributions. Characteristics between women in these groups were compared using *t*-tests or the Wilcoxon signed-rank test, as appropriate. The partial correlation analysis was employed to establish the relationship between the serum IL-17A and TSH levels [corrected for age, gestational week, and body mass index (BMI)]. Multiple linear regression was used to test the association between the TSH level and other parameters. Logistic regression was used to evaluate the risk factors for thyroid disorders. Potential confounding variables, including the gestational week, BMI, age, neutrophil count, lymphocyte count, monocyte count, fasting blood glucose, alanine aminotransferase, and creatinine were controlled in the regression models. When appropriate, natural log-transformed values were used for the analyses. All statistical analyses were performed using the SPSS Statistical Package (version 17.0; Chicago, IL). *P* < 0.05 was considered statistically significant.

## Results

### Clinical Characteristics and Serum IL-17A Levels of Participants

Women with TSH levels ≤ 2.5 mIU/L were similar to those with TSH levels >2.5 mIU/L in terms of the TPOAb and TGAb levels, systolic pressure, diastolic pressure, FBG, ALT, creatinine, white blood cell count, neutrophil count, lymphocyte count, eosinophil count, basophil count, monocyte count, red blood cell count, hemoglobin, and platelet count. However, participants with TSH levels >2.5 mIU/L had a lower mean age (26.43 ± 4.60 vs. 27.88 ± 4.26, *P* < 0.05), lower BMI [20.2 (13.7–28.6) vs. 20.9 (16.0–37.3), *P* < 0.05] and a higher mean gestational week (18.19 ± 2.94 vs. 16.68 ± 2.99, *P* < 0.05) than those with TSH levels ≤ 2.5 mIU/L. Based on the 2017 American Thyroid Association guidelines for thyroid disease during pregnancy, we assigned pregnant women into the euthyroid group (TSH level ≤ 4 mIU/L) and the SCH group (TSH level >4 mIU/L). The SCH subjects had a higher gestational week (18.74 ± 3.02 vs. 16.87 ± 2.93, *P* < 0.05) and a lower age (26.00 ± 4.51 vs. 27.67 ± 4.37, *P* < 0.05). No significant differences in the BMI and other clinical parameters were observed between the two groups (Table 1).

**Table 1:** Clinical characteristics of subjects according to the different TSH levels.

**Variables**	**Total**	**TSH ≤ 2.5**	**TSH > 2.5**	***P*-value**	**TSH ≤ 4**	**TSH > 4**	***P*-value**
*N*	216	124	92	—	163	53	—
TSH^*^	2.02 (0.32–7.01)	1.39 (0.32–2.42)	4.31 (2.67–7.01)	0.000	1.89 (0.32–3.97)	4.92 (4.01–7.01)	0.000
Age (years)	27.26 ± 4.46	27.88 ± 4.26	26.43 ± 4.60	0.018	27.67 ± 4.37	26.00 ± 4.51	0.018
Gestational week (week)	17.33 ± 3.05	16.68 ± 2.99	18.19 ± 2.94	0.000	16.87 ± 2.93	18.74 ± 3.02	0.000
BMI^*^	20.7 (13.67–37.34)	20.9 (16.0–37.3)	20.2 (13.7–28.6)	0.028	20.9 (16.0–37.3)	20.2 (13.7–28.6)	0.446
SBP (mmHg)	115.22 ± 11.42	116.18 ± 11.89	113.94 ± 10.70	0.158	115.84 ± 11.29	113.31 ± 11.72	0.164
DBP (mmHg)	67.89 ± 8.77	67.62 ± 8.62	68.24 ± 8.99	0.612	67.58 ± 8.59	68.83 ± 9.33	0.375
WBC (10^9^/L)	8.34 ± 1.83	8.36 ± 1.86	8.31 ± 1.81	0.838	8.37 ± 1.89	8.24 ± 1.67	0.675
NEUT (10^9^/L)	5.37 ± 2.56	5.62 ± 2.38	5.03 ± 2.75	0.099	5.49 ± 2.52	5.01 ± 2.66	0.239
LYMPH (10^9^/L)	1.48 ± 0.70	1.56 ± 0.63	1.38 ± 0.77	0.068	1.51 ± 0.67	1.41 ± 0.78	0.368
EOS (10^9^/L)	0.07 ± 0.08	0.08 ± 0.08	0.07 ± 0.08	0.392	0.08 ± 0.09	0.06 ± 0.06	0.364
BASO (10^9^/L)	0.01 ± 0.01	0.01 ± 0.01	0.01 ± 0.01	0.254	0.01 ± 0.01	0.01 ± 0.01	0.146
MONO (10^9^/L)	0.35 ± 0.17	0.35 ± 0.15	0.35 ± 0.20	0.907	0.35 ± 0.16	0.34 ± 0.19	0.888
RBC (10^12^/L)	3.95 ± 0.32	3.97 ± 0.30	3.92 ± 0.34	0.356	3.96 ± 0.31	3.92 ± 0.33	0.493
Hb (g/L)	118.10 ± 9.96	118.58 ± 9.13	117.37 ± 11.11	0.418	118.32 ± 9.34	117.36 ± 10.75	0.578
PLT (10^9^/L)	205.99 ± 45.06	202.58 ± 46.57	211.09 ± 42.50	0.206	206.02 ± 46.20	205.91 ± 41.63	0.989
FBG (mmol/L)	4.37 ± 0.45	4.40 ± 0.48	4.33 ± 0.41	0.254	4.37 ± 0.45	4.36 ± 0.45	0.906
ALT (IU/L)	19.03 ± 7.24	19.14 ± 6.80	18.88 ± 7.91	0.911	19.70 ± 8.35	16.98 ± 3.17	0.325
Cr (μmol/L)	41.46 ± 5.36	41.70 ± 5.60	41.15 ± 5.02	0.466	41.55 ± 5.48	41.21 ± 4.98	0.696
FT4 (pmol/L)	14.35 ± 1.55	14.43 ± 1.68	14.24 ± 1.37	0.398	14.32 ± 1.57	14.45 ± 1.50	0.605
TPOAB (IU/mL)^*^	10.10 (5.00–33.50)	10.4 (5.0–33.5)	9.7 (5.0–32.5)	0.188	10.4 (5.0–33.5)	9.7 (5.0–32.5)	0.758
TGAB (IU/mL)^*^	10.00 (0.04–79.80)	10.0 (0.96–73.9)	10.0 (0.04–79.80)	0.523	10.0 (0.96–73.9)	10.0 (0.04–79.80)	0.179

SBP, systolic blood pressure; DBP, diastolic blood pressure; WBC, white blood cells; NEUT, neutrophils; LYMPH, lymphocytes; EOS, eosinophils; BASO, basophils; MONO, monocytes; RBC, red blood cells; Hb, hemoglobin, PLT, platelets; FBG, fasting blood glucose; Cr, serum creatinine.

Data are expressed as the means ± standard deviations (two independent-sample t-test).

* *Data are expressed as the median and range (P-values for Mann-Whitney-test)*.

**Figure 2:**
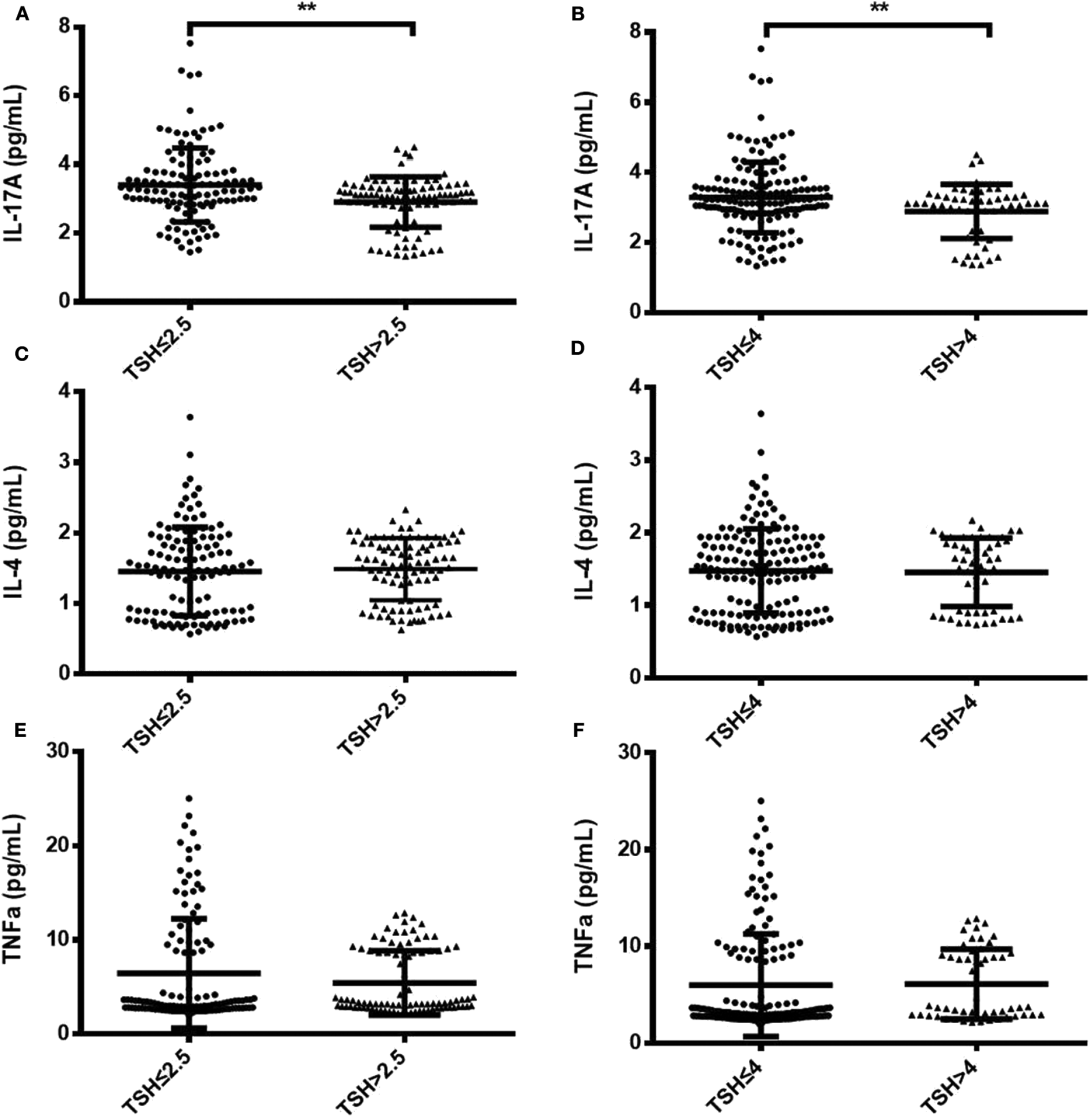
Comparison of serum IL-17A, IL-4, and TNF-α levels between two groups with different TSH levels during the second trimester. Serum IL-17A, IL-4, and TNF-α levels are shown as individual dots for the two cohorts with different TSH cutoff values: TSH level ≤ 2.5 mIU/L (*n* = 124) and TSH level >2.5 mIU/L (*n* = 92) **(A,C,E)**; TSH level ≤ 4 mIU/L (*n* = 163) and TSH level >4 mIU/L (*n* = 53) **(B,D,F)**. The significance levels of the differences between the groups in each subset were analyzed using *t*-tests. Values of *P* considered significant are indicated between two groups (**P < 0.001).

**Figure 3:**
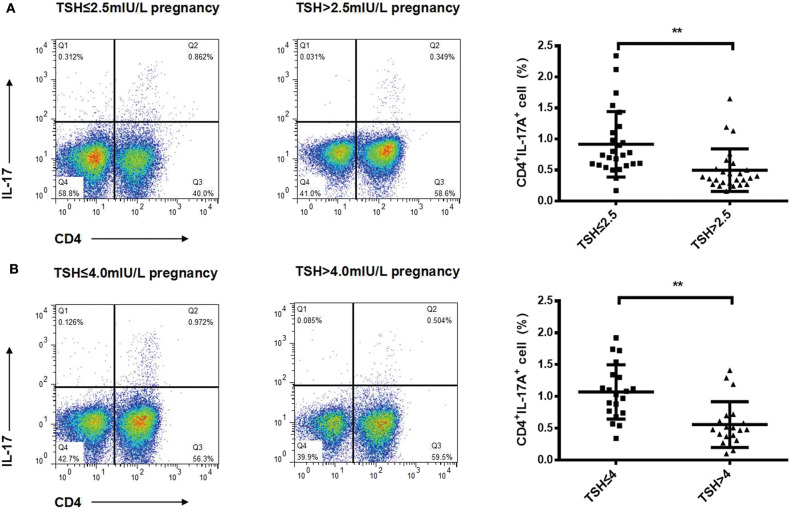
Ratios of Th17 cells decreased progressively with increased TSH concentration in pregnant women during the second trimester. Representative staining of CD4lymphocytes for intracellular IL-17A in pregnant women with TSH levels ≤ 2.5 mIU/L (*n* = 26), >2.5 mIU/L (*n* = 26) **(A)**, ≤ 4 mIU/L (*n* = 20), and >4 mIU/L (*n* = 20) **(B)**. The percentages of CD4^+^ T cells expressing IL-17A are shown as individual dots for the two groups. The significance levels of differences between the groups in each subset were analyzed using the *t*-test (**P < 0.001).

To identify changes in cytokines levels based on serum TSH, we examined the IL-17A, IL-4, and TNF-α level in all participants. The serum IL-4 and TNF-α levels showed no significant differences in women with TSH levels >2.5 mIU/L and those with TSH levels ≤ 2.5 mIU/L nor in women with TSH levels >4 mIU/L and TSH levels ≤ 4 mIU/L. Interestingly, the serum IL-17A level exhibited a significantly decreased trend with increased TSH concentration (*P* < 0.05). A striking result was the statistical difference between women with TSH>2.5 mIU/L and those with TSH ≤ 2.5 mIU/L (2.91 ± 0.73 vs. 3.40 ± 0.90, *P* < 0.01; Figure 2). Because most of the IL-17-producing cells were CD4^+^T cells, we also analyzed the ratio of CD4^+^IL-17A^+^ cells in the peripheral blood lymphocytes of participants. Statistical difference was also found between women with TSH > 2.5 mIU/L and pregnancy-week matched women with TSH ≤ 2.5 mIU/L in the ratio of CD4^+^IL-17A^+^ cells [0.50% (0.16–1.65%) vs. 0.91% (0.17–2.34%), *P* < 0.01; Figure 3]. Similar differences were noted between women with TSH level >4 mIU/L and those with TSH level ≤ 4 mIU/L with respect to the serum IL-17A level (2.87 ± 0.77 vs. 3.29 ± 0.90, *P* < 0.01) and the ratio of CD4^+^IL-17A^+^ cells [0.56% (0.10–1.41) vs. 1.07% (0.34–1.92), *P* < 0.01; Figures 2, 3].

In none of the 53 pregnant women with SCH miscarriage and premature delivery was observed because they were informed to start immediately levothyroxine replacement, while miscarriage and premature delivery rate in those with TSH level ≤ 4 mIU/L were 0.6% (1/163) and 1.2% (2/163), respectively. No significant differences were found in birth weight between pregnant women with SCH and those with TSH level ≤ 4 mIU/L (3344.12 ± 55.06 g, 3322.12 ± 50.16 g, respectively, *p* > 0.05).

### Association Between IL-17A and TSH

Partial correlation analysis demonstrated the strongest correlation between IL-17A and TSH corrected for age, gestational week and BMI (*r* = −0.19, *P* < 0.01) in pregnant women during the second trimester. Remarkably, the serum IL-17A levels were correlated with TSH after stepwise inclusion of additional potential risk factors, such as the gestational week, BMI, age, FBG, ALT, and Creatinine, into the model. Only IL-17A (β = −0.195, *P* = 0.004) and gestational week (β = 0.284, *P* = 0.000) were significant predictors of regression, with TSH as the dependent variable. *T*-test showed that there was no correlation in FT4 and IL17A (*r* = 0.057, *p* = 0.120), FT4 and CD4+IL-17A+ ratio (*r* = −0.144, *p* = 0.350) (data not shown).

We analyzed the risk factors for different TSH cutoff levels using logistic regression with TSH level >2.5 mIU/L and TSH level >4 mIU/L (subclinical hypothyroidism) as dependent variables and IL-17A level as the covariate in fully adjusted models (Table 2). We found that lower serum IL-17A level was a risk factor for TSH level >2.5 mIU/L [odds ratio (OR) = 0.495, 95% confidence interval (CI): 0.336–0.730] and TSH level >4 mIU/L (OR = 0.580, 95% CI: 0.381–0.882) after adjusting for the gestational week, BMI and age during the second trimester (model 1). The ORs were 0.466 (95% CI: 0.309–0.701) for TSH level >2.5 mIU/L and 0.576 (95% CI: 0.378–0.878) for TSH level >4 mIU/L after adjusting for the neutrophil, lymphocyte, and monocyte counts. Interestingly, the ORs for TSH level >2.5 mIU/L (OR = 0.453, 95% CI: 0.298–0.689) and subclinical hypothyroidism (OR = 0.588, 95% CI: 0.385–0.899) were not substantially attenuated by additional adjustment for the FBG, ALT, and Cr (model 3).

**Table 2:** Logistic regression evaluating the association of TSH with IL-17A in fully adjusted models during the second trimester.

	**TSH** **>** **2.5 vs. TSH** **≤** **2.5**		**TSH** **>** **4 vs. TSH** **≤** **4**	
	**OR (95%CI)**	***P***	**OR (95%CI)**	***P***
Model 1	0.495 (0.336–0.730)	0.000	0.580 (0.381–0.882)	0.011
Model 2	0.466 (0.309–0.701)	0.000	0.576 (0.378–0.878)	0.010
Model 3	0.453 (0.298–0.689)	0.000	0.588 (0.385–0.899)	0.013

*Model 1, further adjusted for the gestational week, BMI, and age; Model 2, further adjusted for the neutrophil, lymphocyte, and monocyte counts; Model 3, further adjusted for fasting blood glucose, ALT, and creatinine*.

## Discussion

The identification of a novel Th-cell subset, IL-17-producing Th (Th17) cells, has provided new insight into our understanding of the molecular mechanisms of reproduction. We first observed a significantly negative association between IL-17A level and TSH levels in pregnant women during the second trimester. Moreover, this association is independent of gestational week, BMI, age, neutrophil count, lymphocyte count, monocyte count, FBG, ALT, and Cr.

A successful pregnancy is characterized by immunological tolerance against the semi-allograft fetus; this tolerance is skewed toward a Th2 profile that maintains peripheral tolerance during pregnancy. However, we found that Th1 cells secreting TNF-αand Th2 cells secreting IL-4 were similar between women with different TSH level (Figure 2, Supplementary Figure 1). In addition to TNF-α and IL-4, we also detected some other cytokines, such as IFNγ, IL10, IL22, TGFβ, IL23, and we did not find any significant differences in these cytokines levels between pregnancy-week matched women and normal subjects (Supplementary Figure 2). In our preliminary analysis, we have divided pregnant women into three groups: TSH ≤ 2.5 mIU/ml, 2.5 mI/mlU < TSH ≤ 4 mIU/ml, TSH > 4 mIU/ml. Interestingly we found IL-17A levels in TSH ≤ 2.5 mIU/ml group were significant higher than that in 2.5 mIU < TSH ≤ 4 mIU/ml and TSH > 4 mIU/ml groups, while there was no difference in IL-17A levels between 2.5 mIU < TSH ≤ 4 mIU/ml and TSH > 4 mIU/ml groups (Supplementary Figure 1). IL-17A plays an important role in inducing protective immune responses against extracellular bacteria or fungal pathogens. IL-17A is capable of recruiting neutrophils (20) but also induces neutrophilia and neutrophils are not exclusively protective of infections. Croxatto et al. (21) demonstrated neutrophils in decidua in normal pregnancy are more abundant than that in miscarriage during first trimester. It is possibility that neutrophil expansion mediated by IL17 may be useful for pregnancy. However, we did not find different IL-17A levels in participants with any significant variations in the neutrophil counts between different TSH cutoff groups, indicating that IL-17A may not exclusively play a pivotal role in pro-inflammatory protection. Pongcharoen et al. found that IL-17A was localized in both cyto- and syncytiotrophoblasts of normal-term pregnancies (5). Most of the IL-17-producing cells were CD4^+^T cells (Th17 cells) in healthy pregnancies (22)_._ Wu et al. found that the number of Th17 cells is increased in the peripheral blood of normal pregnancies compared to non-pregnant women (6). The relative numbers of Th17 cells were markedly increased in the decidua compared to peripheral blood during normal pregnancy. Decidual stromal cells (DSCs) recruit peripheral Th17 cells into the decidua by secreting CCL2. The recruited Th17 cells promote proliferation and invasion and inhibit the apoptosis of human trophoblast cells by secreting IL-17A during the first trimester of pregnancy (6). Furthermore, IL-17-treated fibroblasts can up-regulate their angiogenic factors, which are known to promote placental formation (23). IL-17A can also induce the production of matrix metalloproteinase (9), which is important in trophoblast invasion (24). All these data demonstrate a novel role for IL-17A in controlling the maternal-fetal relationship and sustaining normal pregnancy. However in our study, patients with elevated TSH had decreased IL17A and Th17 cells. Negro et al. reported a significantly higher pregnancy loss rate in TPOAb-negative women with TSH concentrations between 2.5 and 5.0 mU/L than in those with TSH concentrations below 2.5 mIU/L (19). A recent study verified that 1.9- and 2.5-fold increased risks of prematurity at <37 and <34 weeks, were observed among women with TSH levels >4.0 mIU/L (25). So we speculated the decreased IL-17A cytokine and CD4^+^IL-17A^+^ cell levels in women with TSH levels >2.5 mIU/L and TSH levels >4 mIU/L may induce higher rates of pregnancy loss and premature delivery. Therefore, in clinical practice, in antibody negative women with SCH and TSH levels >4.0 mIU/L (If pregnancy-specific TSH reference ranges are not available, an upper reference limit of ~4.0 mU/l may be used) may be considered to give oral levothyroxine, and the levothyroxine dose should be adjusted to achieve a TSH value between the lower reference limit and 2.5 mIU/L (14). Therefore, there was little impact of a high TSH level on obstetrical outcomes, including miscarriage, birth weight, and premature delivery due to the rapid levothyroxine supplementation in our study. However, we will verify whether different IL-17A levels have diverse side effects on pregnancy outcome in animals with hypothyroidism in the future.

It is noteworthy that Th17 cells and serum IL-17A levels infiltrating the thyroid gland were significantly increased in Hashimoto's thyroiditis(HT) with higher TGAb compared with controls (26, 27). So we excluded autoimmune thyroid antibody-positive women in order to avoid their confounding effect on IL-17A level in our study. To the best of our knowledge, this study is the first to evaluate the relationship between IL-17A and thyroid function in the absence of thyroid autoimmunity during the second trimester. Most potential confounders, such as age, gestational week, and BMI, were carefully controlled, which limited the possibility of residual confounding effects. We found a negative correlation between the TSH levels and IL-17A by partial correlation (*P* < 0.05). Moreover, a lower IL-17A level was a risk factor for women with TSH levels >2.5 mIU/L and TSH levels >4 mIU/L in fully adjusted logistic regression models. Consistently with our results, serum IL-17A has been inversely associated with serum TSH in hypothyroid HT (26, 28). However positive correlationship between IL-17A and TGAb was verified by Horie et al. They found the titers of TGAb were markedly reduced in iodine-induced autoimmune thyroiditis IL-17^−/−^ NOD-H2h4 mice compared with wild-type NOD-H2h4 mice (29). Therefore, we speculated that when the TSH level was elevated in HT patients with hypothyroidism, it would counteract the effect of increasing IL-17A levels accompanied by TGAb production. Konca et al.'s clinical observation confirmed our hypothesis, they found the euthyroid HT group had the significantly highest levels of IL-17A, whereas the IL-17A levels in the hypothyroid HT patients and control group were similar (28). However, *in vitro* studies we did not observe human TSH had an direct influence on peripheral CD4^+^ T cells differentiate into TH17 cells (Supplementary Figure 3). The indirect interaction between TSH and IL-17-producing CD4^+^ T cells during pregnancy remains unclear. As we all know, many previous studies have confirmed that dendritic cells secreted IL23 or stimulate CD4 cells to secrete TGFβ (30), which can induce TH17 differentiation (31). Although there was no difference in the expression of IL23 and TGFβ in pregnancy-week matched women and normal subjects (Supplementary Figure 2), the data from the serum samples could not confirm there were a direct effect of TSH on the differentiation of TH17 mediated by dendritic cells in the microenvironment. Considering TSH receptor is expressed on leukocytes (32), we will probe this research in the future. Our research found that a low serum IL-17A level during the second trimester is associated with subclinical hypothyroidism. As a matter of fact, our research can't be applied to clinical practice directly. Some studies reported that inhibiting the differentiation of Th17 cells and increasing the differentiation of Treg can induce maternal immune tolerance to the embryo and thereby promoting the development of full-term pregnancy (33). IL-17 therapy is currently limited to animal tumors. Eyerich Kilian show that recombinant IL-17 administered at the site of inoculation of the squamous carcinoma cell line SCCL20 strongly promoted tumor outgrowth in mice (34). But safety and efficacy of IL17 in SCH women is still unclear. Our study reminded clinicians IL-17A acted like a double-edged sword. We speculated the appropriate level of IL-17A is important for pregnant women, lower or higher levels of IL-17A may have a detrimental effect on pregnancy. It's pivotal to enlarge sample size and verify the appropriate range of serum IL-17A in pregnant women.

There are several strength of our current study. First, because chronic infections, gestational diabetes and autoimmune disease can influence IL-17A production, we recruited patients who did not have these diseases. This approach allowed us to better evaluate the immune status and Th1/Th2/Th17 cytokine production for different TSH levels. Second, when we observed that the gestational age was also a predictor for TSH variation, we evaluated the ratio of CD4^+^IL-17A^+^ cells with pregnancy-week matched women in different TSH cutoff groups. Third, our study excluded patients who were receiving any drugs, including thyroid hormone replacement therapy, allowing us to explore correlations between the thyroid hormone levels and immune status in a natural setting. The limitations of our study are as follows. The second trimester is not the best stage to estimate the thyroid autoimmune status as thyroid autoantibody levels decline during pregnancy (35). However, we have not checked TPOAb or TGAb during the first trimester or before pregnancy. Moreover, the study should be extended to populations in the first and third trimesters of pregnancy. We did not evaluate the cytokines and thyroid function levels of the same participants during different phases of pregnancy, and did not compare their cytokine and thyroid function levels before and after pregnancy. We have not evaluated the percent of Tregs, and we could not provide the definitive IL-17A cutoff level for the risk of subclinical hypothyroidism. Due to the difficulty in obtaining blood samples from pregnant women, only some patients among total participants were willing to screen TH17 levels by flow cytometry. Strictly speaking, these patients were not representative of the whole population, and the sample size do not conform to the Gaussian distribution.

In conclusion, our data show a definitive association between the IL-17A and TSH levels in pregnant women during the second trimester. We speculate IL-17A, which plays a critical role in sustaining normal pregnancy may be a risk factor for TSH alteration. However, further studies are needed for verification.

## Data Availability Statement

All datasets generated for this study are included in the article/Supplementary Material.

## Ethics Statement

The medical ethics council of Shanghai Fifth People's Hospital approved this study (Approval No. 081, 2016). Patients and healthy volunteers recruited in this study all provided their written consent to participate in this study.

## Author Contributions

LH, XZ, QY, and CS: conceptualization, methodology, data curation, and writing—original draft preparation. JL and BZ: visualization and supervision. QY and BZ: writing—reviewing and editing. QY, BZ, XH, and XL: software.

## Conflict of Interest

The authors declare that the research was conducted in the absence of any commercial or financial relationships that could be construed as a potential conflict of interest.
