# Supplementary material for: Low Serum IL-17A in Pregnancy During Second Trimester Is Associated With an Increased Risk of Subclinical Hypothyroidism
Source: Front Endocrinol (Lausanne). 2020 May 14;11:298. doi: 10.3389/fendo.2020.00298 (PMC7239996; doi:10.3389/fendo.2020.00298)
Supplement: Supplementary file 1 [file Data_Sheet_1.doc]

B

C

A

Supplementary Figure 1：The expression of cytokines among three groups during second trimester.

The expression of TH17A,IL-4 and TNFα were tested by ELISA during pregnancy. Difference of these cytokine levels were analyzed among three groups：TSH level ≤2.5 mIU/L , TSH level >2.5 mIU/L and TSH≤4 mIU/L, TSH level ＞4 mIU/L. The significance levels of the differences among the groups in each subset were analyzed using t tests. Values of P considered significant are indicated between two groups (**P<0.01).

A

B

C

D


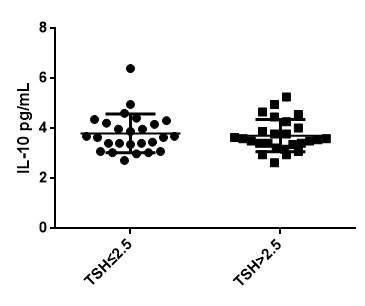

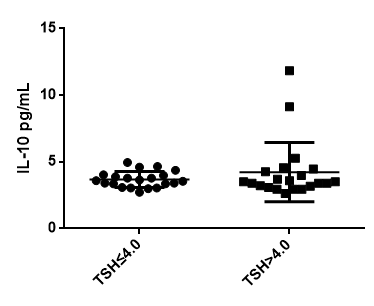


E

F


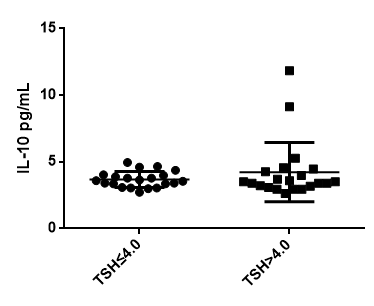


G

H

I

J


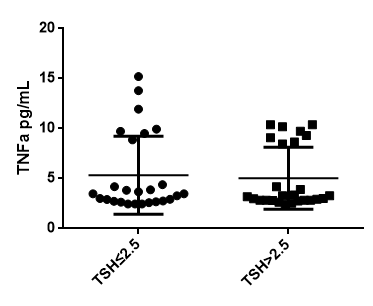

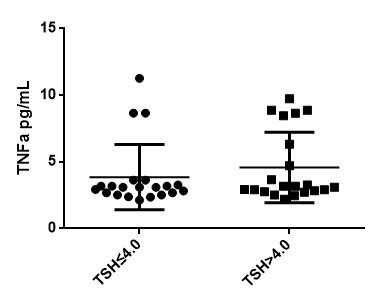


K

L

M

N


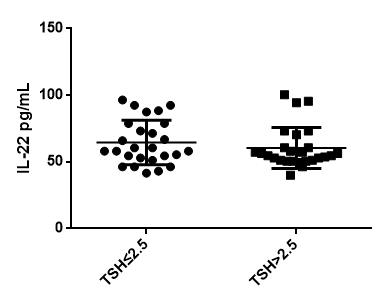

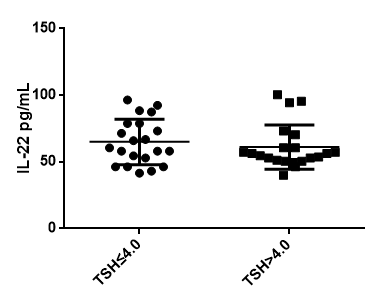


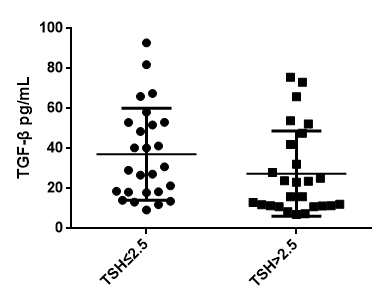

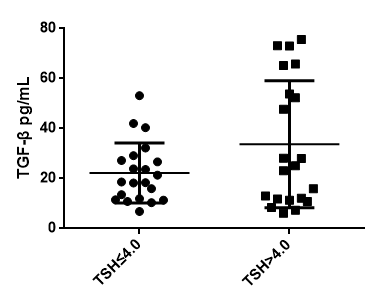


O

P

Supplementary Figure 2：The expression of cytokines between pregnant-week matched two groups during second trimester.

The expression of IL-4,IL-10,IL-23,IL-17A,IFN-γ, TNFα,IL-22 and TGF-β during pregnancy were tested by ELISA. Differences in these cytokines levels were analyzed between two groups matched pregnant-week. IL-4,IL-10,IL-23,IL-17A,IFN-γ, TNFα,IL-22 and TGF-β are shown as individual dots for the two cohorts with different TSH cutoff values: TSH level ≤2.5 mIU/L (n=26) and TSH level >2.5 mIU/L (n=21) (A,C,E,G,I,K,M,O); TSH level ≤4 mIU/L (n=26) and TSH level >4 mIU/L (n=21) (B,D,F,H,J,L,N,P).The significance levels of the differences between the groups in each subset were analyzed using t tests. Values of P considered significant are indicated between two groups (*P<0.05,***P<0.001).

A

B

C

D


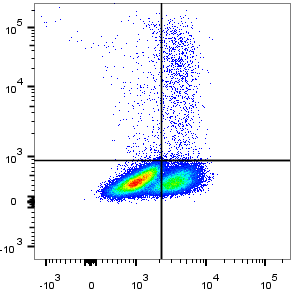

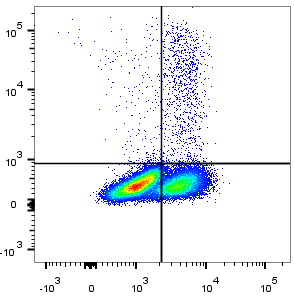

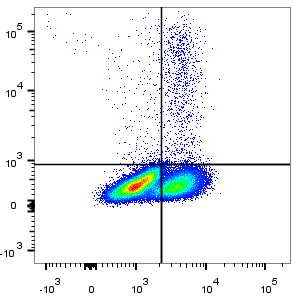

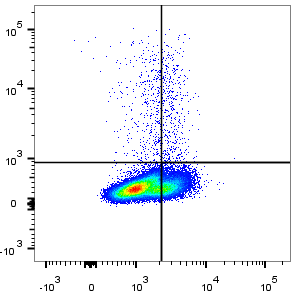


**IL17A-PE**

**2.08%**

**2.19%**

**2.32%**

**0.86%**

**CD4-FITC**

E

Supplementary Figure 3：TSH had no direct influence on peripheral CD4+ T cells differentiate into TH17 cells.

1μg/ml anti-CD3,2μg/ml anti-CD28,10 ng/ml IL-1β,10 ng/ml IL-6,1 ng/ml TGF-β and 10 ng/ml IL-23 were used to promote the proliferation and differentiation of TH17 lymphocyte from normal human peripheral blood . 5 mU/l (B),50 mU/l(C)concentrations of TSH and 100 nM calcitriol (D) were added to culture simultaneously. After four days cells were stained with CD3-Pacific Blue,CD4-FITC,CD8-Percpcy5.5,PE-conjugated anti-IL-17A. Ratios of Th17 cells were screened by flow cytometry(A-D).The percentages of Th17 cell in different groups are shown (E). Compared to control group **P<0.01
